# Clinician decision-making for polymyxin dosing in critically ill patients with renal impairment: a qualitative study

**DOI:** 10.1038/s41598-026-55210-8

**Published:** 2026-06-04

**Authors:** Asha K. Rajan, Vijayanarayana Kunhikatta, Ravindra Prabhu Attur, Souvik Chaudhuri, Vishal Shanbhag, Girish Thunga

**Affiliations:** 1 Department of Pharmacy Practice, Manipal College of Pharmaceutical Sciences, Manipal Academy of Higher Education, Manipal, Karnataka 576104 India; 2 Department of Nephrology, Kasturba Medical College, Manipal Academy of Higher Education, Manipal, Karnataka India; 3 Department of Critical Care Medicine, Kasturba Medical College, Manipal Academy of Higher Education, Manipal, Karnataka 576104 India; 4 Centre for Toxicovigilance and Drug Safety, Manipal College of Pharmaceutical Sciences, Manipal Academy of Higher Education, Manipal, Karnataka 576104 India

**Keywords:** Polymyxin, Renal impairment, Critical care, Qualitative research, Antimicrobial stewardship, Dosing decision-making, Diseases, Health care, Medical research, Nephrology

## Abstract

Polymyxin remain essential last-line agents for the treatment of multidrug-resistant gram-negative infections in critically ill patients. However, dosing polymyxin B and colistin in patients with renal impairment is clinically challenging due to complex pharmacokinetics, fluctuating renal function, toxicity concerns and limited implementation of therapeutic drug monitoring. While pharmacokinetic and guideline-based recommendations exist, little is known about how clinicians navigate these complexities in real-world practice. We conducted an inductive qualitative study using semi-structured interviews with physicians involved in the management of critically ill patients with severe gram-negative infections. Participants were purposively sampled from critical care, nephrology, infectious diseases and general medicine. Interviews explored experiences and decision-making related to polymyxin selection, dosing adjustments, renal dysfunction, renal replacement therapy, safety monitoring and institutional influences. Transcripts were analysed using a constructivist grounded theory approach, following iterative coding, constant comparison and theme development. Coding and analysis were performed using ATLAS.ti software. Reporting adhered to the COREQ checklist. Seventeen physicians were interviewed. Analysis yielded six interrelated themes: 1. Clinical determinants of polymyxin selection, 2. Individualized and adaptive dosing practices, 3. Renal dysfunction and renal replacement therapy-related uncertainty, 4. Safety-driven decision-making, 5. Monitoring, diagnostic, and evidence limitations, and vi. Institutional and system-level influences. Clinicians described polymyxin dosing as a dynamic experience-driven process requiring continuous reassessment and risk–benefit negotiation. Renal function variability, lack of therapeutic drug monitoring, and absence of standardized institutional protocols contributed to cautious and heterogenous dosing practices, particularly in patients receiving dialysis. Polymyxin dosing in critically ill patients with renal impairment is shaped by complex clinical judgement, safety concerns and systemic constraints rather than rigid adherence to dosing algorithms. These findings highlight a gap between pharmacokinetic evidence and bedside practice and underscore the need for pragmatic, context-appropriate dosing guidance, multidisciplinary stewardship support and decision-support tools to optimize polymyxin use in high-risk populations.

**Supplementary Information:**

The online version contains supplementary material available at 10.1038/s41598-026-55210-8.

## Introduction

Sepsis due to multidrug-resistant (MDR) and extensively drug-resistant (XDR) gram-negative bacteria is a growing global threat, particularly in low- and middle-income countries (LMICs) where critical-care resources are limited, and antimicrobial resistance rates are high^1^. In such settings, mortality among patients with carbapenem-resistant infections remains alarming^2^. The polymyxin (polymyxin B and colistin) have thus re-emerged as indispensable salvage options against MDR pathogens such as *Acinetobacter baumannii, Pseudomonas aeruginosa*, and *Klebsiella pneumoniae*^3^. Optimising polymyxin dosing is essential yet highly challenging in patients with renal impairment, a frequent complication of sepsis. These agents exhibit complex pharmacokinetics and narrow therapeutic windows, with efficacy depending on adequate plasma exposure and safety limited by nephrotoxicity^4^. International consensus guidelines do not recommend dose adjustments for polymyxin B in patients with renal impairment^5^, as no consistent correlation has been demonstrated between its clearance and renal function^6,7^. In contrast, the guidelines provide clear dosing instructions for colistin (colistimethate sodium) that specifies dose modifications for patients with renal impairment and those receiving renal replacement therapy (RRT)^5^. However, these recommendations are seldom followed in routine clinical practice due to variability in CMS formulations, complex conversion between international units and milligrams of colistin base activity, and the absence of therapeutic drug monitoring (TDM) to confirm exposure. Consequently, clinical practice remains highly heterogenous, particularly in LMIC intensive care units, where access to dosing tools, monitoring facilities, and dedicated antimicrobial stewardship teams is often limited^8^.

This uncertainty has tangible consequences. Inadequate dosing risks treatment failure, while aggressive regimens may accelerate kidney injury or neurotoxicity^9^. Furthermore, variability in CMS formulations, differing dialysis modalities, and lack of TDM exacerbate inconsistency in clinical outcomes^10^. Although numerous PK/PD studies and simulation models have been published, translating these findings into bedside dosing strategies remains difficult^11,12^. Physicians often rely on individual judgement, institutional norms or anecdotal experience rather than harmonised evidence. Previous qualitative studies on antibiotic decision-making highlight that prescribing is influenced by professional hierarchies, inter-disciplinary communications and organisational culture as much as by pharmacologic rationale^13,14^. However, no qualitative research has yet examined how clinicians specifically navigate the complexities of polymyxin use in renally impaired patients. Understanding this process is critical because physicians are the key decision-makers who balance pharmacologic evidence, renal function dynamics, infection severity and safety considerations when initiating or modifying polymyxin therapy.

Therefore, this study aimed to explore physicians’ experiences and decision-making process regarding the dosing of polymyxin B and colistin in sepsis patients with renal impairment, including those receiving RRT. It sought to identify the clinical, institutional and behavioural factors influencing these choices and to map barriers and facilitators to implementing optimised dosing in real-world ICU practice. Through these insights, the study aims to bridge the gap between pharmacokinetic modelling and bedside practice, informing the development of context-appropriate dosing strategies and stewardship interventions suited to LMIC critical care environments.

## Methodology

### Ethical considerations

The study was approved by the Institutional Ethics Committee (Approval No: 388/2022) and conducted in accordance with the National Ethical Guidelines for Biomedical and Health Research involving Human Participants, and the principles outlined in the Declaration of Helsinki. Participation was voluntary, and written informed consent was obtained from all the physicians after providing them with detailed study information. No financial or other incentives were offered to the participants.

### Study design

The qualitative study employed semi structured interviews analysed using a constructivist grounded theory approach , as described by Charmaz^15^. This approach was selected to facilitate an in-depth exploration of clinicians’ decision-making processes and to allow analytic categories to emerge inductively from participant experiences. While grounded theory principles informed iterative data collection, constant comparison and coding, the primary aim of the study was interpretive thematic exploration rather than the development of a formal theory. Accordingly, the analysis focused on generating conceptually rich themes and identifying relationships between them to construct an exploratory framework of clinical decision-making^15^. This approach was used to enhance analytic depth and conceptual integration rather than to generate a formal grounded theory.

### Development and validation of interview guide

**Fig. 1:**
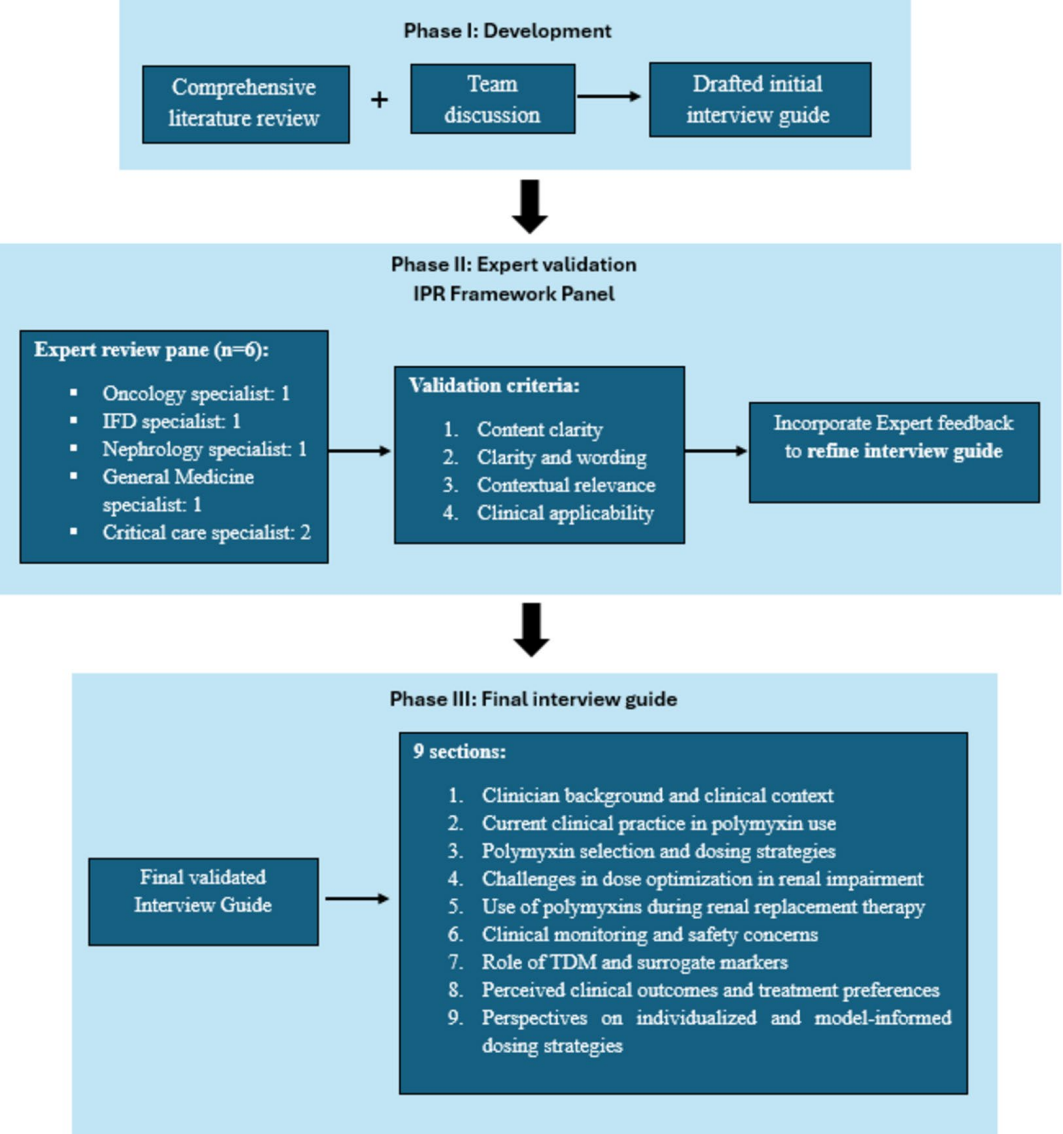
Development and framework of the semi-structured interview guide.

An initial interview guide was developed in English following an extensive review of literature and discussion among the research team. The guide underwent expert validation through the Interview Protocol refinement (IPR) process by a multidisciplinary panel comprising critical care physicians, nephrologists, infectious disease specialists and an oncologist. Based on expert feedback, the guide was refined to ensure content relevance, clarity and contextual appropriateness. The final interview guide included open-ended questions covering i. demographic details of participants, ii. current practices in polymyxin use, iii. challenges in dosing adjustment, iv. dosing considerations in patients receiving RRT, v. clinical monitoring and safety, vi. perceived treatment outcomes, vii. perspectives on individualized and model-informed dosing strategies, and viii. barriers and enablers to implementing optimized dosing. A flowchart on interview guide framework is given in Fig. 1. The complete version of the interview guide is provided in Supplementary file S1. The classification of interview questions is presented in supplementary table 1.

### Participants and recruitment

A purposive sampling strategy was used to identify physicians involved in the management of sepsis across critical care, infectious diseases, nephrology and general medicine departments. Eligible participants were required to have at least five years of clinical experience managing critically ill patients. Invitations were emailed to 30 physicians across various teaching and corporate hospitals of India (Karnataka, Andra Pradesh, Kerala, Pune). Of these, thirteen declined participation, citing lack of expertise in polymyxin dosing, time constraints or unspecified reasons, while seventeen consented to be interviewed. Each invitation included the study background, objectives and consent information. Participation was voluntary, and confidentiality was assured. No compensation was provided.

### Data collection

Interviews were conducted between September and November 2025 using a semi-structured format. All interviews were carried out via audiovisual digital platforms by the primary researcher (AKR) with training in qualitative research, experience in antimicrobial stewardship and clinical pharmacology. Interviews were moderated by a physician (VS), who provided clinical input and probing where required; however, not all interviews required active moderation. No prior professional or personal relationships existed between the interviewer and participants before the study. Participants were informed of the interviewer’s professional background and the study objectives during recruitment and prior to obtaining consent. This transparency was intended to facilitate open discussion while minimizing potential bias related to perceived hierarchy or role expectations Each session lasted between 30 and 70 min (median: 48 min; IQR: 40–58 min). Interviews were audio-recorded with participant permission and transcribed verbatim using artificial intelligence-assisted transcription [Microsoft Teams], followed by manual verification for accuracy by the research team. The interviewer summarized key points at the end of each session to allow participants to confirm and clarify their perspectives. Consistent with grounded theory principles, data collection and analysis occurred concurrently to enhance theoretical sensitivity and iterative refinement of emerging themes. Additional prompts were incorporated as new insights arose during earlier interviews. Field notes were maintained throughout and used to capture contextual observations and preliminary interpretations. Audio files were processed using secure, password-protected systems, and transcripts were de-identified prior to analysis. Access to audio recordings and transcripts was restricted to the research team to ensure confidentiality. Data collection continued until theoretical sufficiency was achieved, defined as the point at which no new conceptual categories, properties or relationships emerged from the data, and existing categories were sufficiently developed and internally coherent. No repeat interviews were conducted thereafter. This was assessed through ongoing concurrent analysis and team discussions during the data collection process.

### Data analysis

Data analysis followed Charmaz’s constructivist grounded theory methodology^15^. Three researchers (AKR, VS, GT) independently read and coded the transcripts, beginning with initial coding to identify concepts grounded in participants’ words. These codes were then grouped into focused codes and subsequently synthesized into subthemes and themes reflecting broader conceptual relationships. As analysis progressed, interpretive coding was undertaken to integrate categories and develop a coherent conceptual framework to explain physicians’ dosing decision processes. Throughout the team engaged in constant comparison, comparing data across participants, codes, and themes, to ensure internal consistency and conceptual depth.

Validation of transcripts was performed using a three-pass-per-tape review, and proof-reading was conducted by two independent researchers to ensure fidelity of transcription (AKR, VS). Transcripts were not returned to participants for member checking. Instead, interpretive validity was ensured through investigator triangulation, peer debriefing, and iterative consensus discussions among the research team. An audit trail documenting coding decisions and theme development was maintained, and constant comparison across transcripts was used to enhance analytic consistency and depth. Data management and coding were performed using ATLAS.ti scientific software development GmbH software (version 22), which facilitated systematic organization and retrieval of codes^16^. The relationship between interview guide sections and the resulting analytic themes is summarized in supplementary table 2.

Through iterative analysis and team-based discussions, a central interpretive construct was identified that integrated the relationships across themes. This core construct conceptualizes polymyxin dosing in critically ill patients with renal impairment as a dynamic, experience-driven process of continuous risk–benefit negotiation under conditions of clinical and systemic uncertainty. Rather than representing a formal grounded theory, this construct informed the development of a conceptual framework that illustrates how clinical, renal, safety, monitoring and institutional factors interact to shape dosing decisions in practice.

### Rigor and trustworthiness

Credibility was ensured through investigator triangulation, iterative coding and reflective memoing during analysis. Dependability was maintained by documenting analytic decisions in an audit trail. Transferability was supported by detailed contextual descriptions of participants and settings, and confirmability was achieved through peer debriefing among the research team to minimize bias and maintain analytic transparency. These strategies collectively supported credibility, dependability, and confirmability of the findings.

### Interviewer characteristics and reflexivity

The research team comprised clinical pharmacists and physicians with experience in critical care, infectious diseases and antimicrobial stewardship. Several team members were familiar with polymyxin prescribing practices in ICU settings, which informed the study design and interpretation of findings. The primary interviewer (AKR) had prior training in qualitative interviewing and was not involved in the direct clinical management of participants, which helped reduce potential power differentials. The physician moderator (VS) contributed clinical insights during selected interviews to enhance depth and contextual understanding. Participants were aware of the professional backgrounds of the research team and the purpose of the study. Reflexive discussions were conducted within the research team throughout data collection and analysis to ensure that interpretations remained grounded in participant narratives rather than influenced by prior assumptions.

## Results

**Fig. 2:**
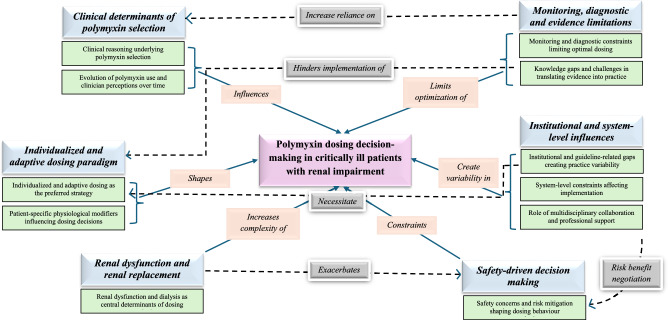
Conceptual network map illustrating factors influencing polymyxin dosing decision-making in critically ill patients with renal impairment.

Seventeen physicians involved in the management of critically ill patients with severe gram-negative infections were interviewed. Participants represented diverse clinical backgrounds, including critical care medicine, nephrology, infectious diseases and general medicine with a wide range of clinical experience. Interviews explored clinicians’ perspectives on polymyxin selection, dosing practices, renal dysfunction-related challenges, safety considerations, monitoring constraints and institutional influences. Inductive analysis yielded six interrelated analytic themes with eleven subthemes, reflecting the complex, multi-layered nature of polymyxin dosing decision-making in critically ill patients with renal impairment. The relationships between codes, subthemes and overarching themes identified through inductive analysis are illustrated in the conceptual network map , which provides a visual representation of how individual concepts interact to form higher-order themes rather than presenting them as isolated categories. These relationships are further elaborated in supplementary table 3 and visually summarized in Fig. 2, which helps to trace the progression from raw data to thematic structure. These themes are not independent domains but interrelated components of a broader decision-making process, as described below.

### Participant characteristics

The study included 17 physicians (13 male, 4 female), with ages ranging from 33 to 61 years and clinical experience ranging from 6 to 35 years. Most participants were trained in critical care medicine, followed by nephrology, infectious diseases and general medicine. Detailed demographic and professional characteristics are summarized in supplementary table 4.

#### Theme 1: Clinical determinants of polymyxin selection

Clinicians described polymyxin selection as a context-sensitive clinical decision, informed by infection characteristics, microbiological considerations, and experiential knowledge. Rather than relying on rigid treatment algorithms, participants emphasized pragmatic reasoning shaped by patient presentation, infection source and prior clinical exposure to polymyxin in their clinical practice.

### Infection source

The anatomical source of infection emerged as a primary determinant in selecting between polymyxin B and colistin. Clinicians consistently articulated a source-based heuristic, wherein colistin was preferentially considered for urinary tract and renal-source infections, while polymyxin B was favoured for bloodstream and pulmonary infections. This distinction was described as evolving from institutional practice norms and personal experience rather than formal guideline directives. Several participants highlighted that this source-based differentiation helped simplify decision-making in complex ICU settings, particularly when rapid treatment initiation was required. Others acknowledged that while this approach was commonly followed, it was not universally applied and could vary depending on patient-severity and co-morbid conditions.

If is something related to urine or pyelonephritis, we usually go with colistin. Anything related to blood or lungs, we prefer polymyxin B.[PFN-40]

For bloodstream infections and pneumonia, polymyxin B is what we generally think of.[PFID-33]

However, some participants emphasized that infection source alone was not sufficient to dictate drug choice and needed to be interpreted alongside overall clinical severity and patient stability.

Yes, source matters, but sometimes the patient condition overrides everything else. [PMN-45]

If the patient is very sick, we don’t strictly go by source alone. [PMCCM-47]

#### Resistance patterns

Participants frequently framed polymyxin use within the broader context of rising antimicrobial resistance. Increasing rates of multidrug-resistant and hospital-acquired infections were described as reducing therapeutic options and necessitating reliance on polymyxin as last-line agents. Clinicians noted that resistance patterns, particularly carbapenem resistance, often triggered consideration of polymyxin early in the treatment course. However, participants differed in how strongly resistance patterns alone dictated polymyxin initiation. Some viewed microbiological data as central to decision-making whereas others emphasized that resistance findings had to be weighed against clinical stability and anticipated toxicity.

Most of the infections we see in the ICU now are MDR, so polymyxin come into the picture more often. [PFGP-38]

Ideally, we wait for cultures, but in sick patients we sometimes start empirically. [PMCCM-51]

Resistance patterns guide us, but clinical judgement still plays a role. [PMCCM-52]

#### Clinician experience

Clinical experience emerged as a subtle but influential factor in polymyxin selection. Several clinicians developing comfort with specific polymyxin over time, shaping their prescribing preferences. Perceived differences in dosing complexity, ease of administrations, and familiarity influenced drug choice, particularly between polymyxin B and colistin. While some participants explicitly acknowledged that these preferences were experience-driven rather than evidence-based, others viewed experiential knowledge as essential in navigating the uncertainties surrounding polymyxin use.

Somehow, I feel polymyxin B dosing is slightly easier compared to colistin. It is less clumsy. [PFGP-39]

Based on experience, you develop comfort with one drug over the other. [PMCCM-40a]

It may not be evidence-based every time, but experience matters in these cases. [PMGP-61]

What you have seen working before influences your choice. [PFID-33]

#### Theme 2: Individualized and adaptive dosing paradigm

Participants uniformly emphasized that polymyxin dosing in critically ill patients must be individualized and continuously adapted, rather than following fixed or standardized regimens. Dosing decisions were described as dynamic processes shaped by patient-specific physiological factors such as illness severity, organ dysfunction, fluid status, weight and evolving clinical response, with renal function considered as one of the several influencing factors.

### Patient-specific factors

Clinicians highlighted the heterogeneity of critically ill patients as a key reason for individualized dosing. Factors such as illness severity, organ dysfunction, fluid status and evolving clinical trajectory were cited as influencing dose selection. Participants described adapting dosing strategies in response to changes in patient condition rather than adhering to predefined protocols.

For each and every patient, we calculate the renal clearance and dose accordingly. [PMN-48]

There is no one-size-fits-all approach in these patients. [PMCCM-38]

Some clinicians described tailoring doses based on bedside assessment rather than strict numerical calculations.

Sometimes it’s not just numbers; it’s how the patient looks clinically. [PMCCM-51]

#### Weight and renal function as dosing input

Weight and renal function assessment were consistently identified as central parameters in dosing decisions. However, clinicians acknowledged practical challenges in obtaining accurate weight measurements in ICU settings, leading to reliance on estimated or predicted body weight. Similarly, renal function assessment was complicated by rapidly changing creatinine values in critically ill patients. Participants varied in how rigorously these parameters were applied, with some describing systematic calculations and others relying more on approximate adjustments. While renal function was an important parameter in dose adjustment, clinicians primarily discussed it as part of broader patient-specific assessment rather than as an isolated source of uncertainty.

Weight and GFR are the main parameters we look at when deciding the dose. [PMGP-42]

Often we don’t get actual weight, so we rely on estimated or predicted weight. [PMN-48]

Renal function assessment was similarly described as complex, with clinicians noting rapid fluctuations in creatinine and urine output.

Renal function is not stable in these patients. [PMCCM-51]

Creatinine today may not reflect what is happening tomorrow. [PMCCM-48]

#### Dynamic dose adjustment

Dosing was described as an iterative and ongoing process rather than a one-time calculation. Clinicians reported adjusting doses based on clinical response, renal function trends and perceived toxicity risk. Rather than fixed maintenance regimens, polymyxin dosing was framed as requiring frequent reassessment.

Day-to-day improvements are seen, and dosage adjustments are made accordingly. [PMGP-61]

As the patient improves or worsens, the dose also changes.[PFN-40]

Some participants emphasized the need for close monitoring and frequent reassessment, while others acknowledged that such adjustments were sometimes limited by workload or system constraints.

Ideally, we should reassess every day, but practically it doesn’t always happen. [PFID-33]

#### Theme 3: Renal dysfunction and renal replacement therapy

Renal dysfunction was consistently described as a distinct source of structural and operational uncertainty in polymyxin dosing, particularly in the context of fluctuating renal function and renal replacement therapy. Participants emphasized that both baseline renal impairment and dynamic changes in renal function during critical illness complicated dose selection and adjustment. RRT, in particular, was viewed as an area where clinical practice relied heavily on pragmatism rather than standardized guidance. Unlike general patient-specific adjustments, renal impairment, especially in patients receiving RRT, introduced uncertainty related to drug clearance, dialysis modality and timing, which was not easily addressed through standard dose individualization.

#### Acute kidney injury and renal variability

Clinicians described AKI and fluctuating renal function as common in critically ill patients receiving polymyxin, often rendering static dosing recommendations inadequate. Participants highlighted the challenge of interpreting creatinine trends in the context of rapidly evolving clinical status, fluid shifts and organ support. Several clinicians noted that renal function often changed faster than dosing adjustments could be implemented, increasing uncertainty and prompting cautious decision-making.

Renal function keeps fluctuating in these patients, so whatever dose you decide today may not be applicable tomorrow. [PMN-45]

AKI makes things unpredictable. You calculate based on today’s creatinine, but tomorrow it may be completely different. [PMCCM-40b]

Others emphasized that renal variability often led to conservative dosing, particularly in patients already showing signs of kidney injury.

#### Dialysis modality and timing

Clinicians described dialysis timing as an operational consideration that strongly influenced how polymyxin were administered. Most clinicians reported adopting simplified strategies, such as administering polymyxin after intermittent hemodialysis, to minimize confusion and reduce the risk of dosing errors. Importantly, these practices were often justified on practical or safety grounds rather than on firm pharmacokinetic evidence.

In general, we give all drugs post-HD, including polymyxin. [PFID-33]

Even if the drug is not dialyzable, giving it after dialysis avoids any confusion or human error. [PMCCM-49]

However, some participants acknowledged that this approach may not be optimal in all situations and reflected a lack of confidence in available dosing guidance.

Whether post-dialysis dosing is ideal or not, I’m not sure, but practically that’s what we do. [PMN-45]

#### Dialysis-related and RRT-specific uncertainty

Dosing during continuous RRT was described as particularly challenging. Clinicians expressed uncertainty regarding drug clearance, filter adsorption and appropriate dose adjustments during CRRT. Several participants highlighted the absence of clear, practical recommendations as a major limitation. While renal function contributed to dose individualization, clinicians primarily approached it as one component of a broader patient-centred assessment, rather than as a standalone determinant.

During CRRT, dosing always becomes confusing because there is no clear guidance. [PFGP-39]

We don’t really know how much drug is being removed in CRRT, so dosing becomes guesswork. [PMGP-42]

Some clinicians reported relying on experience or specialist inputs when managing patients on RRT, while others admitted to avoiding dose escalation due to safety concerns.

#### Theme 4: Safety-driven decision-making

Safety considerations were deeply embedded in polymyxin prescribing and frequently shaped both initial dosing and subsequent dose adjustments. Participants described toxicity concerns as an ever-present influence, particularly with pre-existing renal impairment or prolonged ICU stays.

### Nephrotoxicity concerns

Fear of nephrotoxicity emerged as a dominant theme influencing clinician behaviour. Participants described heightened vigilance when prescribing polymyxin, often prioritizing renal safety even in the setting of severe infection.

We are always worried about kidney injury when using polymyxin, especially in critically ill patients . [PMN-48]

Once renal function starts worsening, we immediately think about whether polymyxin is contributing. [PMCCM-41]

Some clinicians acknowledged that concerns about nephrotoxicity sometimes led to underdosing or reluctance to escalate therapy.

Sometimes the fear of nephrotoxicity makes us hesitant to increase the dose, even if the infection is severe. [PMGP-42]

### Neurotoxicity concerns

Neurotoxicity was also recognized as a clinically relevant adverse effect, though it was discussed less frequently that nephrotoxicity. Participants described monitoring for neurological symptoms, particularly in patients receiving higher doses or prolonged therapy.

Neurotoxicity is something we do keep in mind, especially with prolonged use. [PFGP-39]

If patients develop confusion or neurological symptoms, we become more cautious with dosing. [PMCCM-51]

Variation was noted in how strongly neurotoxicity influenced dosing decisions, with some clinicians viewing it as secondary concern compared to renal toxicity.

Neurotoxicity is there, but renal toxicity worries us more. [PMGP-61]

### Risk–benefit negotiation

Clinicians frequently framed polymyxin dosing as a balancing act between achieving microbiological control and minimizing harm. Decisions were described as individualized and context-dependent with clinicians weighing infection severity, patient prognosis and toxicity risk.

Sometimes you have to balance between treating the infection aggressively and not harming the patient further . [PMGP-42]

It is always a trade-off-how much risk you are willing to take for infection control. [PFID-33]

Several participants emphasized that this negotiation evolved over time, particularly as patient response and organ function changed.

Initially you may be aggressive, but later you reassess based on how the patient is doing. [PMCCM-40a]

While clinicians rarely explicitly described underdosing as a concern, their accounts suggested an underlying awareness of the trade-off between minimizing toxicity and achieving effective antimicrobial exposure.

#### Theme 5: Monitoring, diagnostic and evidence limitations

Participants consistently described systemic limitations that constrained optimal polymyxin dosing, many of which reflect resource and infrastructure constraints commonly encountered in LMIC critical care settings. These limitations were viewed as external to individual clinical judgement but nevertheless influential in shaping practice.

### Limited access to therapeutic drug monitoring

TDM was widely acknowledged as theoretically beneficial but largely unavailable in routine practice. As a result, clinicians relied on surrogate markers and clinical response to guide dosing. The absence of TDM was frequently attributed to limited availability of specialized laboratory infrastructure, a constraint particularly relevant in resource- limited settings.

Therapeutic drug monitoring would be ideal, but practically it is not available for us. [PMCCM-47]

Some participants expressed frustration that the lack of TDM limited confidence in dosing decisions.

If we had TDM, we would be more confident about whether we are underdosing or overdosing. [PMN-45]

#### Evidence-practice gap

Clinicians described difficulty translating available evidence into bedside practice, particularly in patients with renal dysfunction or on RRT. Guidelines were often perceived as insufficiently detailed or not reflective of real-world ICU populations.

Most of what we do is based on literature and experience . [PMCCM-40a]

The evidence is there but applying it exactly in these patients is difficult. [PFID-33]

Several participants emphasized reliance on experiential knowledge in the absence of robust, context-specific evidence.

At the end of the day, experience plays a big role. [PMN-45]

#### Diagnostic delays

Delayed microbiological results were described as necessitating empirical decision-making often before pathogen-specific information data was available. Participants noted that early dosing decisions were frequently made under uncertainty. This reliance on empirical therapy further reinforced cautious dosing strategies,

By the time culture results come, treatment has already started. [PMCCM-51]

Initial decisions are mostly empirical, especially in sick patients. [PFGP-39]

#### Theme 6: Institutional and system-level influences

Beyond individual clinical judgement, institutional and system-level factors played a significant role in shaping polymyxin dosing practices, often reflecting resource availability and organizational structures typical of LMIC healthcare settings.

### Protocol gaps

Participants consistently highlighted the absence of standardized institutional protocols for polymyxin dosing, particularly in patients with renal impairment or on dialysis. This lack of formal guidance contributed to inter-clinician variability.

We don’t really have a fixed institutional dosing strategy for polymyxin. [PMCCM-51]

It depends a lot on who is treating the patient. [PFGP-38]

Some clinicians expressed a desire for institutional policies to reduce uncertainty and variability.

Having a clear protocol would definitely help. [PMN-48]

#### Resource constraints

These constraints were frequently linked to infrastructure limitations, including restricted access to monitoring tools and equipment, which are more commonly encountered in resource-limited healthcare environments.

Availability of drugs and monitoring facilities is a limitation. [PMN-48]

Even weighing beds are not always available, so weight-based dosing becomes difficult. [PMCCM-38]

#### Multidisciplinary collaboration

Despite institutional constraints, multidisciplinary collaboration was widely viewed as a facilitator of safer and more consistent polymyxin use. Inputs from infectious disease specialists, nephrologists and clinical pharmacists were described as valuable in navigating complex dosing decisions.

Inputs from infectious disease specialists definitely help in decision-making. [PMGP-42]

Having pharmacists involved improves dosing accuracy and confidence. [PFGP-39]

Some clinicians noted that multidisciplinary discussions were particularly helpful in complex renal cases.

For dialysis patients, we usually discuss with nephrology and ID before deciding the dose. [PMCCM-40b]

**Fig. 3:**
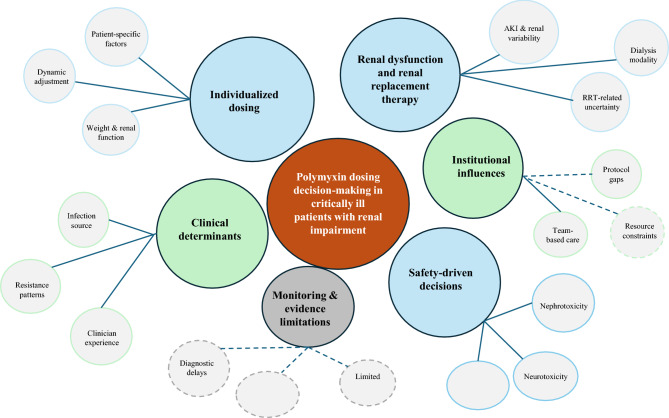
Conceptual thematic framework illustrating polymyxin dosing decision-making in critically ill patients with renal impairment.

To further synthesize these findings, Fig. 3 presents a thematic framework that integrates the identified themes and highlights their interconnections within the broader decision-making process.

#### Central interpretive process underlying polymyxin dosing decision-making

Across the six themes, clinicians consistently described polymyxin dosing as a dynamic and iterative process shaped by competing clinical priorities, uncertainty and contextual constraints. A unifying analytic construct emerged from the data: polymyxin dosing as an iterative process of risk–benefit negotiation under conditions of renal and system-level uncertainty. This process reflects how clinicians continuously balance the need for effective infection control against the risk of toxicity, particularly nephrotoxicity, while accounting for fluctuating renal function, dialysis-related variability, and limitations in monitoring and institutional guidance.

Each of the identified themes contributes to this central process. Clinical determinants (Theme 1) inform initial treatment selection, while individualized and adaptive dosing (Theme 2) reflects ongoing adjustments based on patients’ response. Renal dysfunction and RRT (Theme 3) introduce significant uncertainty, shaping dosing decisions and prompting cautious strategies. Safety-driven decision-making (Theme 4) represents the core tension within this process, with toxicity concerns frequently influencing dose modification or restraint. Monitoring and evidence limitations (Theme 5) further constrain decision-making by limiting objective feedback, reinforcing reliance on clinical judgement. Finally, institutional and system-level influences (Theme 6) define the practical boundaries within which these decisions are made. Together, these interacting domains illustrate that polymyxin dosing is not a linear or protocol-driven process, but a continuously negotiated clinical practice shaped by uncertainty, experience and contextual realities.

## Discussion

**Fig. 4:**
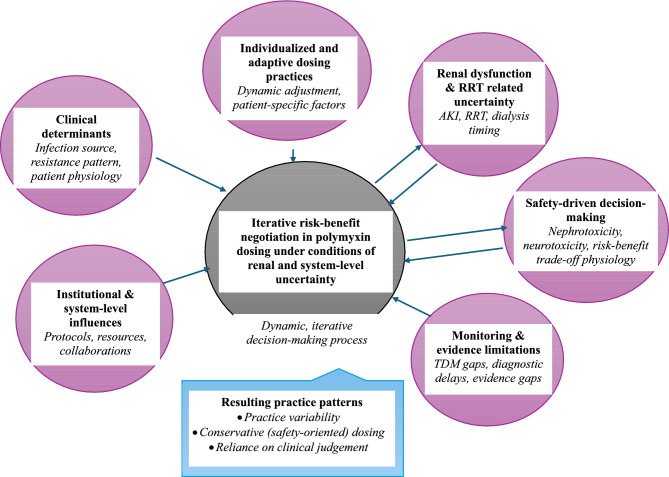
Conceptual framework illustrating factors influencing polymyxin dosing decision-making in critically ill patients with renal impairment.

This qualitative study provides an in-depth, theoretically informed interpretive analysis of clinicians’ perspectives on polymyxin dosing in critically ill patients with renal impairment, highlighting how dosing decisions are shaped by a complex interplay of clinical reasoning, renal-related uncertainty, safety concerns, monitoring limitations, and institutional context. Rather than following standardized algorithms, clinicians described polymyxin dosing as an iterative process of risk–benefit negotiation under conditions of renal and system-level uncertainty, informed by experience and continuously adapted to evolving clinical contexts. These findings add important contextual insight to the growing pharmacokinetic and stewardship literature on polymyxin, particularly by illustrating how clinicians interpret and apply evidence within the constraints of real-world intensive care practice. Rather than validating existing models, this study highlights the contextual, behavioural and system-level factors that shape dosing decisions at the bedside. To examine convergence and variation in perspectives across participants, a matrix map was constructed (Supplementary table 5), which provides a cross-case comparison of how individual clinicians engaged with each theme. Figure 4 presents a conceptual framework illustrating how the identified domains interact to shape polymyxin dosing decision-making, positioning the process as an iterative risk–benefit negotiation under conditions of clinical and system-level uncertainty. Unlike the thematic framework, this model provides an integrative, process-oriented representation of decision-making, highlighting how multiple interacting factors converge to influence dosing practices in real-world settings.

Clinicians in this study consistently described polymyxin selection as a context-driven and experience-informed process, shaped by infection characteristics, resistance patterns and prior clinical familiarity, rather than strict adherence to guideline-driven pathways. The commonly reported preference for colistin in urinary tract and renal-source infections and polymyxin B in bloodstream and pulmonary infections reflects pragmatic bedside reasoning that aligns with pharmacokinetic principles, particularly differences in renal handling and tissue distribution between the two agents^5,17,18^. Previous pharmacokinetic studies have demonstrated that polymyxin B undergoes predominantly non-renal clearance, whereas colistin exposure is more closely linked to renal function through its prodrug colistimethate sodium, which may partially explain clinicians’ source-based preferences^19,20^. However, our findings also highlight variability in how consistently these heuristics are applied. Some clinicians emphasized that infection source alone was insufficient to dictate drug choice, particularly in patients with severe illness or rapidly evolving organ dysfunction. This variability underscores how clinicians actively adapt and reinterpret available evidence in response to patient complexity and uncertainty, highlighting a gap between theoretical recommendations and bedside reasoning^10^.

A central finding of this study is the strong emphasis clinicians place on individualized and adaptive dosing strategies. Participants uniformly rejected fixed dosing regimens, describing polymyxin dosing as an iterative process that evolves with patient-specific physiological factors (e.g., organ function, severity of illness and hemodynamic status), renal function and clinical response. This aligns with population pharmacokinetic and pharmacodynamic studies that have demonstrated wide interpatient variability in polymyxin exposure, particularly in critically ill patients with altered volume of distribution, hypoalbuminemia and organ dysfunction^21,22^. At the same time, clinicians acknowledged significant practical barriers to implementing fully individualized dosing, including challenges in obtaining accurate body weight and interpreting fluctuating renal markers. Although dosing frameworks emphasize individualized approaches, our findings demonstrate that implementation is often shaped by pragmatic constraints, clinical judgement, and feasibility considerations. Clinicians frequently rely on approximation and bedside assessment, reflecting the complexity of translating structured dosing recommendations into dynamic ICU environments^23–25^.

Renal impairment and RRT emerged as distinct contributors to structural uncertainty, extending beyond routine dose individualization and introducing challenges related to drug clearance, dialysis practices and limited guidance. Clinicians described AKI and rapidly changing renal function as undermining confidence in static dosing recommendations, often prompting conservative approaches. This finding is consistent with PK studies showing that creatinine-based estimates may poorly reflect true drug clearance in critically ill patients, particularly during dynamic phases of illness^4,21^. Dosing during RRT was described as especially challenging, with clinicians frequently relying on pragmatic strategies such as post-dialysis administration to reduce operational complexity and perceived risk. While such practices may simplify workflow, they also underscore the absence of clear, clinically actionable guidance for polymyxin dosing during intermittent and continuous dialysis modalities. Despite the availability of some evidence, clinicians described persistent uncertainty in applying this knowledge in practice, reinforcing the reliance on simplified, experience- based strategies in high-risk clinical scenarios^22,26,27^.

These findings highlight several actionable targets for improving clinical practice. first, decision-support tools tailored to RRT settings may help reduce uncertainty by integrating key variables such as dialysis modality (intermittent vs continuous), timing of drug administration relative to dialysis, and evolving renal function. Second, clinicians emphasized the need for simplified frameworks that incorporate minimum essential data elements, including dialysis modality, session timing and clinical trajectory, rather than relying solely on static creatinine-based estimates. Finally, the complexity of dosing in RRT underscores the need for clear multidisciplinary ownership, with collaborative input from infectious disease specialists, nephrologists and clinical pharmacists to support consistent and evidence-informed decision-making.

Safety-driven decision-making was a prominent theme, with nephrotoxicity and neurotoxicity exerting a strong influence on dosing choices. Clinicians consistently described fear of renal injury as a key factor constraining dose escalation, sometimes even in the context of severe infection. While, this cautious approach reflects the well-documented nephrotoxic potential of polymyxin, it also introduces the risk of suboptimal antimicrobial exposure, which may contribute to treatment failure and, potentially, the emergence of resistance^28,29^. Importantly, participants did not always explicitly frame conservative dosing as compromising efficacy; however, their narratives reflected an implicit tension between achieving adequate infection control and minimizing harm. This highlights a disconnect between safety-driven clinical heuristics and PK/PD informed exposure targets, which emphasize the need for sufficient drug exposure to optimize microbiological outcomes^30,31^. In practice, clinicians often appeared to prioritize short-term safety, particularly in patients with existing renal dysfunction, even when this may have resulted in more conservative dosing strategies. This tension underscores a critical challenge in polymyxin stewardship, where clinicians must navigate competing priorities in the absence of real-time exposure data or robust decision-support tools. Addressing this gap may require the development of pragmatic dosing framework that better integrate safety considerations with exposure-based targets, particularly for high-risk populations.

Participants consistently described the absence of practical decision-support tools, including TDM, as a major constraint shaping reliance on clinical judgment. This finding is consistent with the current state of polymyxin stewardship, where TDM is recommended in principle but remains unavailable or impractical in many settings^4,32^. In the absence of exposure data, clinicians relied heavily on clinical response and toxicity monitoring, reinforcing the experiential nature of dosing decisions. In addition, clinicians described difficulty translating available evidence into bedside practice, particularly for patients with renal impairment or on RRT. This perceived evidence-practice gap reflects broader concerns in the literature regarding the applicability of PK/PD studies conducted in controlled settings to heterogenous ICU populations^33^. Diagnostic delays further compounded uncertainty, necessitating empirical decision-making early in the course of illness.

Institutional and system-level factors played a critical role in shaping polymyxin dosing practices. The absence of standardized institutional protocols contributed to inter-clinician variability, with dosing decisions often dependent on individual experience and comfort. Resource constraints, including limited access to monitoring tools and basic infrastructure, further influenced practice, particularly in high-acuity environments. Despite these constraints, multidisciplinary collaboration emerged as an important facilitator of safer and more confident dosing decisions. Inputs from infectious disease specialists, nephrologists and clinical pharmacists was viewed as particularly valuable in complex renal cases. This finding aligns with stewardship literature emphasizing the role of multidisciplinary teams in optimizing antimicrobial use, especially for high-risk agents such as polymyxin^24,34^.

While several findings in this study reflect challenges specific to LMIC critical care settings, particularly limited access to TDM, infrastructure constraints, and absence of standardized institutional protocols, the underlying clinical decision-making process appear to be broadly applicable across diverse healthcare contexts. The central process identified in this study, characterized by iterative risk–benefit negotiation under uncertainty, is likely inherent to polymyxin prescribing in critically ill patients regardless of setting. However, the extent and manifestation of uncertainty may differ, with resource limitations amplifying reliance on clinical judgement in LMIC environments. In contrast, settings with greater access to diagnostic tools, TDM and structured protocols may mitigate some of these constraints but are unlikely to eliminate the fundamental tension between efficacy and safety. These findings highlight both the context-dependent nature of system-level constraints and the broader relevance of clinician-driven decision-making processes, supporting the applicability of this conceptual framework beyond LMIC settings while acknowledging differences in implementation.

**Fig. 5:**
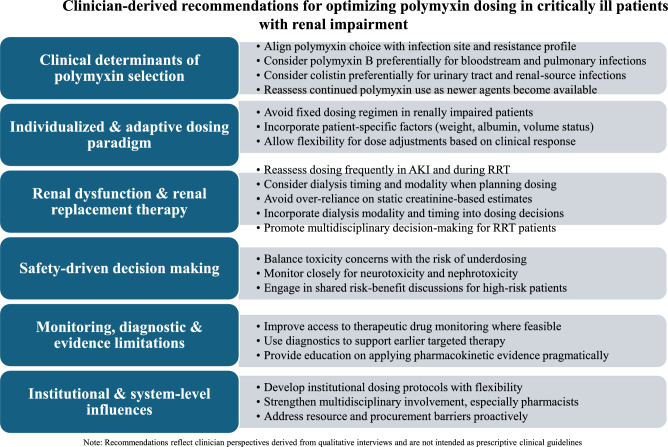
Clinician-derived recommendations for optimizing polymyxin dosing in critically ill patients with renal impairment.

By adopting an inductive qualitative approach, this study provides nuanced insights into real-world polymyxin dosing practices that are not readily captured through quantitative or pharmacokinetic studies alone. The use of matrix mapping and a conceptual thematic framework allowed for systematic comparison across participants while preserving the contextual richness of individual perspectives. The findings extend beyond existing PK/PD and stewardship literature by providing insight into how clinicians navigate uncertainty, integrate fragmented evidence, and make context-sensitive decisions in rela-world settings. This qualitative perspective captures dimensions of clinical reasoning and system-level influence that are not accessible through pharmacokinetic or quantitative approaches alone. Based on clinicians’ perspectives, a set of practice-oriented recommendations for optimizing polymyxin dosing was developed (Fig. 5), which translates qualitative insights into actionable considerations for clinical practice rather than reiterating thematic findings. These findings should be interpreted as contextual insights into clinician decision-making processes, rather than prescriptive guidance for clinical practice.

### Strengths

This study has several strengths that enhance the credibility and relevance of its findings. The use of an inductive qualitative design enabled an in-depth exploration of clinicians’ real-world decision-making processes related to polymyxin dosing in critically ill patients with renal impairment; an area where quantitative evidence and guideline clarity remain limited. By prioritizing participants’ perspectives, the study captured nuanced clinical reasoning and contextual factors that are not readily accessible through pharmacokinetic or outcomes-based studies alone. The inclusion of clinicians from multiple relevant specialities, including critical care, nephrology and infectious diseases, provided a broad and multidisciplinary view of polymyxin use in complex clinical scenarios. This diversity strengthened the richness of the data and allowed identification of shared challenges as well as variation in practice across disciplines. The inclusion of participants from LMIC settings provides important insight into how resource constraints influence antimicrobial decision-making, a perspective that remains underrepresented in existing literature. Reflexive interviewing, probing and saturation-oriented sampling helped ensure depth and completeness of the findings. Finally, by situating clinicians’ experiential insights alongside existing pharmacokinetic, stewardship and dosing literature, this study provides complementary qualitative evidence that can inform future guideline development, model-informed dosing strategies and antimicrobial stewardship interventions.

### Limitations

This study has limitations that should be considered when interpreting its findings. The study was conducted at a limited number of institutions and involved a small sample of physicians, which may restrict the transferability of findings to other healthcare settings. The relatively small sample size, although appropriate for qualitative inquiry, may limit the breadth of perspective captured. While thematic depth and theoretical sufficiency were achieved, the findings may not fully represent all variations in clinical practice across different settings. Although participants represented diverse specialities involved in the management of critically ill patients, perspectives from other stakeholders, such as nursing staff, microbiologists and hospital administrators, were not included and may have provided additional insight into system-level influences on polymyxin use. Interviews may be subject to recall bias or social desirability bias, particularly when discussing complex prescribing decisions involving high-risk antimicrobials. However, this limitation was mitigated through the use of open-ended questioning and probing to encourage reflective and candid responses. The study focused on clinicians’ decision-making processes rather than directly observing prescribing behaviour. As a result, discrepancies may exist between reported practices and actual bedside behaviour. Future studies incorporating observational methods or mixed-methods designs may help further contextualize these findings. Finally, although TDM and pharmacokinetic modeling were discussed by participants, the study did not evaluate the implementation or effectiveness of these strategies in practice. Consequently, the findings should be interpreted as reflecting perceived challenges and needs rather than definitive assessments of specific dosing interventions.

### Implications for practice and future research

The findings suggest that efforts to optimize polymyxin dosing should move beyond guideline dissemination alone and address the contextual realities of critical care practice. Development of pragmatic, institution-specific dosing protocols, integration of model-informed dosing tools and expansion of multidisciplinary stewardship support may help bridge the gap between evidence and practice.

In particular, the uncertainty surrounding dosing during RRT highlights the need for structured decision-support approaches, including simplified dosing frameworks that incorporate key clinical variables such as dialysis modality, timing of administration and patient-specific factors. Embedding these tools within routine ICU workflows, supported by coordinated input from infectious disease specialist, nephrologists and clinical pharmacists, may improve consistency and confidence in dosing decisions. Future research should focus on validating such approaches in real-world settings and exploring these findings in larger, multi-center and mixed-method studies to enhance generalizability and clinical applicability.

## Conclusions

This qualitative study highlights the complexity of polymyxin dosing decision-making in critically ill patients with renal impairment, revealing a practice landscape shaped by individualized clinical judgement, renal-related uncertainty, safety concerns and institutional constraints. Clinicians described polymyxin dosing as a dynamic and adaptive process, often relying on experiential knowledge and multidisciplinary collaboration in the absence of standardized protocols, TDM, or robust, context-specific evidence. The findings underscore a clear gap between pharmacokinetic and stewardship recommendations and their practical implementation at the bedside, particularly in patients with fluctuating renal function or receiving RRT. Safety considerations, especially nephrotoxicity, strongly influenced prescribing behaviour, frequently leading to conservative dosing strategies that may compromise optimal antimicrobial exposure. Together, these insights emphasize the need for pragmatic, clinician-friendly approaches to polymyxin dosing that account for real-world constraints. Institutional dosing protocols, model-informed decision-support tools, enhanced multidisciplinary stewardship involvement and context-appropriate monitoring strategies may help bridge the gap between evidence and practice. By elucidating how clinicians navigate uncertainty in high-risk prescribing scenarios, this study contributes valuable qualitative evidence to inform future guideline development, stewardship initiatives and research aimed at optimizing polymyxin use in critically ill populations. These findings are intended to inform understanding of clinical decision-making processes and highlight areas for improvement, rather than to provide direct clinical dosing recommendations. Larger studies are needed to further validate and translate these insights into practice.

## Supplementary Information

Supplementary Information 1.

Supplementary Information 2.

Supplementary Information 3.

Supplementary Information 4.

Supplementary Information 5.

Supplementary Information 6.

## Data Availability

All relevant data are included within the manuscript and its supporting materials. The data generated and analysed during this study consist of anonymized interview transcripts from participating clinicians. Due to the qualitative nature of the study and to protect participant confidentiality, full transcripts are not publicly available but may be made available from the corresponding author upon reasonable request and with appropriate ethical approvals. Any additional information related to the study may be obtained from the institutional ethics committee of Kasturba Medical College and Kasturba Hospital , email: [iec.kmc@manipal.edu](mailto:iec.kmc@manipal.edu) .

## Abbreviations

AKI: Acute kidney injury
MDR: Multi-drug resistant
MODS: Multi organ dysfunction syndrome
RRT: Renal replacement therapy
ICU: Intensive care unit
TDM: Therapeutic drug monitoring
CRRT: Continuous renal replacement therapy
CKD: Chronic kidney disease
PK/PD: Pharmacokinetic/pharmacodynamic
