# Supplementary material for: Clinician decision-making for polymyxin dosing in critically ill patients with renal impairment: a qualitative study
Source: Sci Rep. 2026 Jun 4;16:25420. doi: 10.1038/s41598-026-55210-8 (PMC13473469; doi:10.1038/s41598-026-55210-8)
Supplement: Supplementary file 1 [file 41598_2026_55210_MOESM1_ESM.docx]

**Supplementary file S1: Final interview guide for qualitative research**

1. Could you please tell me about yourself and your current clinical role?

*Probes:*

- What is your professional qualification and designation?
- How long have you been involved in the care of critically ill patients?
- Approximately how often do you manage sepsis patients with renal impairment in your clinical practice?
- Over time, have you noticed any patterns or recurring challenges in the management of such patients?

2. Could you describe how you usually approach the use of polymyxin B and colistin in sepsis patients with renal impairment?

*Probes:*

- What clinical or patient-related factors influence your choice between polymyxin B and colistin ?
- To what extent do institutional, national or international guidelines influence your dosing decisions ?
- Could you describe the typical loading and maintenance doses you use for these agents?
- How do you adjust doses based on renal function or changes in clinical status?
- Have you observed a preference among colleagues for one polymyxin over the other? What do you think drives that preference?

3. What challenges do you commonly face when adjusting polymyxin doses in patients with renal impairment?

*Probes*

- Have you encountered conflicting recommendations from guidelines, consultants or institutional protocols ?
- In your experience, have you noticed differences in efficacy or safety between different brands, particularly for colistimethate sodium?
- Can you describe a situation where you modified a polymyxin dose due to changes in renal function ?
- What clinical cues or laboratory parameters guided your decision in that situation?

4. How do your approach polymyxin dosing in sepsis patients who are receiving renal replacement therapy?

*Probes:*

- How do you tailor dosing based on the type of renal replacement therapy (intermittent hemodialysis, sustained low-efficiency dialysis, or continuous renal replacement therapy)?
- Do you modify the timing of polymyxin administration in relation to dialysis sessions? What informs this decision ?
- How do you manage situations where a scheduled dose overlaps with a dialysis session?
- Have you had concerns regarding filter adsorption or altered drug clearance during RRT? How do you address these issues ?
- Do you routinely administer supplemental doses after dialysis? What factors guide this decision?

5. How do you monitor the effectiveness and safety of polymyxin therapy in your patients?

*Probes:*

- What clinical and laboratory parameters do you routinely monitor during therapy?
- How frequently are these parameters assessed and what determines the monitoring frequency?
- What has been your experience with nephrotoxicity in patients receiving polymyxin B or colistin?
- Have you observed differences in toxicity profiles between the two agents? Have you encountered neurotoxicity or other non-renal adverse effects? how were these identified and managed?

6. Have you used therapeutic drug monitoring or any surrogate markers to guide polymyxin dosing decisions?

*Probes:*

- If yes, what motivated you to use therapeutic drug monitoring?
- How helpful were the results in guiding real-time dose adjustments?
- If not, what barriers have limited the use of therapeutic drug monitoring in your setting?
- Are there alternative clinical or laboratory markers you rely on instead?

7. Based on your experience, have you noticed any differences in clinical outcomes between colistin and polymyxin B in sepsis patients with renal impairment?

*Probes:*

- Have you observed differences in patient recovery, such as resolution of septic shock or improvement in acute kidney injury
- What factors do you believe contributed to those outcomes?

8. In your opinion, how strongly do dosing decisions influence recovery trajectories in these patients? What is your perspective on individualized or model-informed dosing strategies for polymyxins?

*Probes:*

- Do you see value in population pharmacokinetic-guided or model-informed dosing approaches in your ICU setting?
- What practical challenges do you foresee in implementing such approaches in routine clinical practice ?
- What kind of training, tools or institutional support would be required to make this feasible?

9. What barriers or facilitators influence the optimization of polymyxin dosing in your ICU setting?

*Probes:*

- Are there workflow-related challenges that make timely dose adjustment difficult?
- How does the presence of infectious disease specialists or clinical pharmacists influence your decisions?
- Have you encountered resistance from peers or institutional policies when initiating or modifying polymyxin therapy?
- During ICU rounds, do you have easy access to relevant patient data needed for dosing decisions?
- Have you faced challenges related to drug preparation, dilution or infusion times or product labelling?

10. Finally, based on your experience, what suggestions would you offer to improve polymyxin dosing in critically ill patients with renal impairment?

*Probes:*

- Would you be willing to support educational or stewardship initiatives for clinicians or nursing staffs?
- How cost-effective do you consider therapeutic drug monitoring for polymyxins in your setting?
- Is there anything else from clinical experience that you feel to share ?
