# Supplementary material for: Clinician decision-making for polymyxin dosing in critically ill patients with renal impairment: a qualitative study
Source: Sci Rep. 2026 Jun 4;16:25420. doi: 10.1038/s41598-026-55210-8 (PMC13473469; doi:10.1038/s41598-026-55210-8)
Supplement: Supplementary file 2 [file 41598_2026_55210_MOESM2_ESM.docx]

**Supplementary Table 1: Classification of interview questions used in the semi-structured interview guide**

| **Type of question** | **Explanation of type of question** | **Examples from the present study** |
| --- | --- | --- |
| **Introductory questions** | Open-ended questions intended to initiate the interview, build rapport, and elicit general clinical experience without directing responses toward specific outcomes. | *Over time, have you noticed any patterns or recurring challenges in the management of such patients?* |
| **Transition questions** | Questions that bridge general clinical experience to the specific focus of the study, guiding participants toward discussion of polymyxin use and dosing considerations. | *What clinical or patient-related factors influence your choice between polymyxin B and colistin ?* |
| **Key questions** | Core questions directly aligned with the research objectives, designed to explore clinical reasoning, dosing decision-making, and contextual factors influencing polymyxin use in renally impaired critically ill patients. | *Finally, based on your experience, what suggestions would you offer to improve polymyxin dosing in critically ill patients with renal impairment?* |
| **Probing questions** | Follow-up questions used flexibly to clarify, elaborate or deepen participant responses, allowing exploration of reasoning and contextual details. | *Do you modify the timing of polymyxin administration in relation to dialysis sessions? What informs this decision ?* |
| **Reflective questions** | Questions that encourage participants to reflect on challenges, uncertainties and evolving practices related to polymyxin dosing and decision-making. | *Have you faced challenges related to drug preparation, dilution or infusion times or product labelling?* |
| **Closing questions** | Questions intended to provide closure to the interview and allow participants to share additional insights not previously discussed. | *What kind of training, tools or institutional support would be required to make this feasible?* |
