# Supplementary material for: Clinician decision-making for polymyxin dosing in critically ill patients with renal impairment: a qualitative study
Source: Sci Rep. 2026 Jun 4;16:25420. doi: 10.1038/s41598-026-55210-8 (PMC13473469; doi:10.1038/s41598-026-55210-8)
Supplement: Supplementary file 3 [file 41598_2026_55210_MOESM3_ESM.docx]

**Supplementary Table 2: Mapping of interview guide sections to analytic themes**

| **Sl No.** | **Interview questions** | **Clinical determinant guiding polymyxin selection** | **Individualized and adaptive dosing paradigm** | **Renal dysfunction and renal replacement therapy** | **Safety-driven decision-making and risk-benefit negotiation** | **Monitoring, diagnostic and evidence-related limitations** | **Institutional, system-level and collaborative influences** |
| --- | --- | --- | --- | --- | --- | --- | --- |
| 1 | Participant background and clinical experience | X |  |  |  |  | X |
| 2 | Patterns and challenges in managing sepsis with renal impairment | X | X | X | X |  | X |
| 3 | Approach to polymyxin B and colistin selection | X | X |  | X |  | X |
| 4 | Dosing strategies and dose adjustment in renal impairment |  | X | X | X |  | X |
| 5 | Polymyxin dosing during renal replacement therapy |  | X | X | X |  | X |
| 6 | Monitoring effectiveness and safety of polymyxins |  |  | X | X | X |  |
| 7 | Experience with nephrotoxicity and neurotoxicity |  |  |  | X | X |  |
| 7 | Use and feasibility of therapeutic drug monitoring |  |  |  |  | X | X |
| 8 | Clinical outcomes and perceived effectiveness | X | X |  | X |  |  |
| 9 | Perspectives on model-informed or PK-guided dosing |  | X |  |  | X | X |
| 10 | Barriers and facilitators to dose optimization |  | X | X | X | X | X |
| 11 | Recommendations to improve polymyxin dosing practices | X | X | X | X | X | X |
