# Supplementary material for: Clinician decision-making for polymyxin dosing in critically ill patients with renal impairment: a qualitative study
Source: Sci Rep. 2026 Jun 4;16:25420. doi: 10.1038/s41598-026-55210-8 (PMC13473469; doi:10.1038/s41598-026-55210-8)
Supplement: Supplementary file 4 [file 41598_2026_55210_MOESM4_ESM.docx]

**Supplementary Table 3: Themes, subthemes and exemplar quotations from clinician interviews**

| **Theme** | **Subtheme** | **Exemplar quotations** |
| --- | --- | --- |
| **Clinical determinants of polymyxin selection** | Infection source | *“If it is something to urine or pyelonephritis, we usually go with colistin. Anything related to blood or lungs, we prefer polymyxin B”.* |
|  | Resistance patterns | *“Now we are seeing more and more multidrug-resistant infections, especially hospital-acquired ones, which is why polymyxins come into the picture”.* |
|  | Clinician experience | *“Somehow, I feel polymyxin B dosing is slightly easier compared to colistin. It is less clumsy”.* |
| **Individualized and adaptive dosing paradigm** | Patient-specific factors | “*For each and every patient, we calculate the renal clearance and then decide the dose accordingly*” |
|  | Weight and renal function | *“Based on the patient’s weight and the GFR, the dose should be adjusted rather than following a fixed dose”.* |
|  | Dynamic adjustment | *“Dat-to-day improvements are seen, and dosage adjustments are made accordingly”.* |
| **Renal dysfunction and renal replacement therapy** | AKI and renal variability | *“Renal function keeps fluctuating in these patients, so the dose cannot be the same throughout”* |
|  | Dialysis modality and timing | *“In general, we give all drugs post-HD. Even if it is not dialyzable, it avoids any possible impact or human error”.* |
|  | RRT-related uncertainty | *“During dialysis, dosing always becomes confusing because there is no clear guidance”.* |
| **Safety-driven decision-making** | Nephrotoxicity concerns | *“We are always worried about kidney injury when using polymyxins, especially in critically ill patients”.* |
|  | Neurotoxicity concerns | *“Neurological complications are something we keep in mind, particularly when higher doses are considered”.* |
|  | Risk-benefit negotiation | *“Sometimes we have to balance between treating the infection aggressively and not harming the patient further”.* |
| **Monitoring, diagnostic and evidence limitations** | Limited access to TDM | *“Therapeutic drug monitoring is ideal, but practically it is not available for us”.* |
|  | Evidence-practice gap | *“Most of what we do is based on literature and experience, but applying it exactly is difficult”.* |
|  | Diagnostic delays | *“By the time culture results come, we have already started treatment based on clinical judgment”.* |
| **Institutional and system-level influences** | Protocol gaps | *“We don’t really have a fixed institutional dosing strategy for polymyxins”.* |
|  | Resource constraints | *“Availability of drugs, monitoring facilities and even weighing beds can be a limitation”.* |
|  | Multidisciplinary collaboration | *“Having inputs from infectious disease specialists and clinical pharmacists definitely helps in decision-making”.* |
