# Supplementary material for: Clinician decision-making for polymyxin dosing in critically ill patients with renal impairment: a qualitative study
Source: Sci Rep. 2026 Jun 4;16:25420. doi: 10.1038/s41598-026-55210-8 (PMC13473469; doi:10.1038/s41598-026-55210-8)
Supplement: Supplementary file 5 [file 41598_2026_55210_MOESM5_ESM.docx]

**Supplementary Table 4: Characteristics of included participants**

| **Participant** | **Age** | **Gender** | **Years of experience** | **Highest degree** | **Background** |
| --- | --- | --- | --- | --- | --- |
| Physician 1 | 33 | Female | 6 | DM (Infectious Diseases) | ID |
| Physician 2 | 61 | Male | 35 | MD (General Medicine) | GM |
| Physician 3 | 40 | Female | 12 | DM (Nephrology) | NPH |
| Physician 4 | 39 | Female | 13 | MD (General Medicine), IDCCM | CCM |
| Physician 5 | 48 | Male | 15 | DM (Nephrology) | NPH |
| Physician 6 | 38 | Male | 11 | FNB (Critical care medicine) | CCM |
| Physician 7 | 42 | Male | 15 | MD (General Medicine) | GM |
| Physician 8 | 51 | Male | 25 | MD (General Medicine), FCICM | CCM |
| Physician 9 | 41 | Male | 15 | DM (Critical care medicine) | CCM |
| Physician 10 | 40 | Male | 11 | FNB (Critical care medicine) | CCM |
| Physician 11 | 44 | Male | 15 | MD (Anesthesia), IDCCM, IFCCM | CCM |
| Physician 12 | 40 | Male | 10 | DM (Critical care medicine), EDIC | CCM |
| Physician 13 | 52 | Male | 25 | FRCP | CCM |
| Physician 14 | 38 | Female | 10 | MD (General Medicine) | GM |
| Physician 15 | 45 | Male | 11 | DM (Nephrology) | NPH |
| Physician 16 | 49 | Male | 15 | IDCCM | CCM |
| Physician 17 | 47 | Male | 20 | IDCCM, ICCM | CCM |

CCM: Critical Care Medicine; EDIC: European Diploma in Intensive Care Medicine; FNB: Fellowship of National Board; FRCP: Fellow of the Royal College of Physicians; GM: General Medicine; NPH: Nephrology; DM: Doctor of Medicine; IDCC: Indian diploma in critical care medicine; IFCCM: Indian fellowship in critical care medicine; ID: Infectious disease; GM: General medicine

Mean age: 42 (33-61) years

Median years of experience: 15 (6-35) years
