# Supplementary material for: Clinician decision-making for polymyxin dosing in critically ill patients with renal impairment: a qualitative study
Source: Sci Rep. 2026 Jun 4;16:25420. doi: 10.1038/s41598-026-55210-8 (PMC13473469; doi:10.1038/s41598-026-55210-8)
Supplement: Supplementary file 6 [file 41598_2026_55210_MOESM6_ESM.docx]

**Supplementary table 5: Matrix map illustrating cross-case patterns in clinician perspectives on polymyxin dosing**

| **Participant** | **Clinical determinants guiding polymyxin selection** | **Individualized and adaptive dosing paradigm** | **Renal dysfunction and renal replacement therapy** | **Safety-driven decision-making and risk-benefit negotiation** | **Monitoring, diagnostic, and evidence-related limitations** | **Institutional and system-level influences** |
| --- | --- | --- | --- | --- | --- | --- |
| PFN-40 | Emphasized infection patterns and rising MDR burden influencing polymyxin choice | Supported individualized dosing based on patient condition | Recognized renal impairment as a complicating factor | Expressed caution regarding toxicity in critically ill patients | Limited discussion of formal monitoring strategies | Noted reliance on institutional norms and expert opinion |
| PMGP-61 | Favoured protocol-based selection of polymyxins | Preferred standardized dosing within institutional policy | Acknowledged dialysis considerations with limited dose adjustment | Safety considerations guided conservative dosing | Relied on literature rather than real-time monitoring | Strong emphasis on institutional policy and protocol development |
| PMN-48 | Differentiated polymyxin choice by infection source and organ involvement | Viewed polymyxin B dosing as easier and more adaptable | Considered renal function central to dosing decisions | Balanced toxicity concerns with clinical necessity | Limited access to monitoring tools acknowledged | Highlighted ICU workflow and nursing practices |
| PFGP-39 | Relied on prior clinical experience for polymyxin selection | Individualized dosing informed by clinical judgement | Identified dialysis timing as relevant but inconsistently applied | Demonstrated cautious risk-benefit negotiation | Limited familiarity with TDM and PK models | Noted dependence on specialist input and system constraints |
| PMCCM-41 | Highlighted MDR patterns influencing polymyxin use | Supported adaptive dosing in unstable patterns | Recognized fluctuating renal function as complicating dosing | Expressed concern about nephrotoxicity | Acknowledged evidence-practice gap | Reported lack of standardized protocols |
| PMCCM-38 | Considered polymyxins are salvage therapy | Favoured patient-specific dose modification | Noted challenges during CRRT | Safety concerns limited dose escalation | Limited feasibility of therapeutic monitoring | Cited resource constraints |
| PMGP-42 | Emphasized clinical severity in polymyxin selection | Individualized dosing viewed as necessary | Renal impairment increased dosing uncertainty | Safety concerns influenced conservative practice | Limited awareness of PK-guided dosing | Highlighted variability across departments |
| PMCCM-51 | Relied on resistance patterns for polymyxin choice | Supported flexible dosing approaches | Dialysis status guided dosing decisions | Risk-benefit trade-offs explicitly acknowledged | Diagnostic delays affected optimization | Emphasized multidisciplinary consultation |
| PFGP-38 | Noted evolving use of polymyxins over time | Adaptive dosing preferred over fixed regimens | Renal dysfunction frequently prompted reassessment | Toxicity concerns shaped dosing restraint | Monitoring limitations constrained precision | Absence of institutional guidance noted |
| PMCCM-52 | Contextualized polymyxin choice by infection site | Individualization guided by clinical response | Identified AKI as a major dosing determinant | Safety considerations dominated decision-making | Limited access to evidence-based tools | System level constraints highlighted |
| PFID-33 | Favoured polymyxin B for non-urinary infections | Viewed individualized dosing as essential | Dialysis timing inconsistently incorporated | Balanced toxicity risk with treatment urgency | Limited exposure to TDM | Relied on senior clinician input |
| PMCCM-40a | Emphasized experiential learning in drug selection | Supported clinician-driven dose tailoring | Renal impairment necessitated frequent adjustments | Conservative approach due to toxicity concerns | Difficulty applying literature to practice | Institutional variability acknowledged |
| PMCCM-40b | Considered polymyxins a last-line option | Adaptive dosing used in complex cases | Renal replacement therapy increased complexity | Risk-benefit negotiation ongoing | Evidence gaps limited confidence | Lack of formal protocols reported |
| PMCCM-44 | Used polymyxins selectively based on clinical context | Favoured individualized dosing strategies | Renal dysfunction influenced dose reductions | Safety concerns limited aggressive dosing | Limited diagnostic support noted | Dependence on multidisciplinary teams |
| PMN-45 | Noted preference shifts with emerging alternatives | Individualization seen as necessary but challenging | Renal impairment complicated dosing decisions | Toxicity risk drove cautious practice | Limited familiarity with PK models | System constraints affected implementation |
| PMCCM-47 | Emphasized clinician judgement in polymyxin choice | Flexible dosing approach adopted | Dialysis variability increased uncertainty | Safety considerations influenced hesitation | Monitoring gaps highlighted | Institutional practices shaped decisions |
| PMCCM-49 | Integrated infection characteristics in drug selection | Individualized dosing preferred in critically ill | Renal dysfunction central to dosing strategy | Risk-benefit considerations explicitly stated | Evidence translations challenges noted | Absence of standardized guidance emphasized |

*Note: Cells summarize participants’ engagement with each theme based on analytic synthesis of interview data and do not represent frequency counts.*
